# Supplementary material for: MetaRibo-Seq measures translation in microbiomes
Source: Nat Commun. 2020 Jun 29;11:3268. doi: 10.1038/s41467-020-17081-z (PMC7324362; doi:10.1038/s41467-020-17081-z)
Supplement: Supplementary file 10 — Supplementary Data 7 [file 41467_2020_17081_MOESM10_ESM.zip › File2/Confidence_High_Taxonomy/9244_out.krona.html]

Javascript must be enabled to view this page.

members
magnitude
magnitudeUnassigned
count
unassigned
taxon
rank

9244\_out

5

5

SRS018984\_contig\_number\_contig-80\_4443.172638SRS019693\_contig\_number\_contig-100\_5141.5142SRS024388\_contig\_number\_13607SRS049959\_contig\_number\_contig-100\_5127.93644SRS144537\_contig\_number\_contig-100\_6723.148265
